# Effects of Maltodextrin–Fructose Supplementation on Inflammatory Biomarkers and Lipidomic Profile Following Endurance Running: A Randomized Placebo-Controlled Cross-Over Trial

**DOI:** 10.3390/nu16183078

**Published:** 2024-09-12

**Authors:** Stefano Righetti, Alessandro Medoro, Francesca Graziano, Luca Mondazzi, Serena Martegani, Francesco Chiappero, Elena Casiraghi, Paolo Petroni, Graziamaria Corbi, Riccardo Pina, Giovanni Scapagnini, Sergio Davinelli, Camillo Ricordi

**Affiliations:** 1 Department of Cardiology, Fondazione IRCCS San Gerardo dei Tintori, 20900 Monza, Italy; stefano.righetti@irccs-sangerardo.it; 2 Department of Medicine and Health Sciences “V. Tiberio”, University of Molise, 86100 Campobasso, Italy; alessandro.medoro@unimol.it (A.M.); giovanni.scapagnini@unimol.it (G.S.); 3 Biostatistics and Clinical Epidemiology, Fondazione IRCCS San Gerardo dei Tintori, 20900 Monza, Italy; francesca.graziano@unimib.it; 4 Bicocca Bioinformatics Biostatistics and Bioimaging Center B4, School of Medicine and Surgery, University of Milan-Bicocca, 20126 Milan, Italy; 5 Sport Service Mapei, Sport Nutrition, 21057 Olgiate Olona, Italy; luca.mondazzi@mapeisport.it; 6 School of Clinical Nutrition, University of Milan, 20126 Milan, Italy; 7 Department of Biotechnology and Life Science, University of Insubria, 21100 Varese, Italy; serena.martegani@uninsubria.it; 8 Equipe Enervit Srl, Scientific Research Unit of Enervit Spa, 20126 Milan, Italy; assistenza.reactionhub@gmail.com (F.C.); elena.casiraghi@unipv.it (E.C.); p.petroni@enervit.com (P.P.); r.pina@enervit.com (R.P.); 9 Department of Public Health, Experimental and Forensic Sciences, University of Pavia, 27100 Pavia, Italy; 10 Department of Translational Medical Sciences, University of Naples “Federico II”, 80138 Naples, Italy; graziamaria.corbi@unina.it; 11 Cell Transplant Center, Diabetes Research Institute, Miller School of Medicine, University of Miami, Miami, FL 33136, USA; cricordi@med.miami.edu

**Keywords:** carbohydrate, running, high-intensity, endurance, inflammation, inflammatory biomarkers, interleukin-6, ω-3 index, AA/EPA ratio

## Abstract

Background: Managing metabolism for optimal training, performance, and recovery in medium-to-high-level endurance runners involves enhancing energy systems through strategic nutrient intake. Optimal carbohydrate intake before, during, and after endurance running can enhance glycogen stores and maintain optimal blood glucose levels, influencing various physiological responses and adaptations, including transitory post-endurance inflammation. This randomized trial investigates the impact of a high-dose 2:1 maltodextrin–fructose supplementation to medium-to-high-level endurance runners immediately before, during, and after a 15 km run at 90% VO_2max_ intensity on post-exercise inflammatory stress. Methods: We evaluated inflammatory biomarkers and lipidomic profiles before the endurance tests and up to 24 h after. We focused on the effects of high-dose 2:1 maltodextrin–fructose supplementation on white blood cell count, neutrophil number, IL-6, cortisol, and CRP levels, as well as polyunsaturated fatty acids, ω-3 index, and AA/EPA ratio. Results: This supplementation significantly reduced inflammatory markers and metabolic stress. Additionally, it may enhance the post-activity increase in blood ω-3 fatty acid levels and reduce the increase in ω-6 levels, resulting in a lower trend of AA/EPA ratio at 24 h in the treated arm. Conclusions: Adequate carbohydrate supplementation may acutely mitigate inflammation during a one-hour endurance activity of moderate-to-high intensity. These effects could be beneficial for athletes engaging in frequent, high-intensity activities.

## 1. Introduction

Managing metabolism to optimize training, performance, and recovery for medium- and high-level runners involves understanding and strategically enhancing the energy systems. This includes optimizing nutrient intake and efficient utilization of metabolic sources to support sustained energy production during training sessions and races and ensuring adequate recovery between workouts [1,2]. During endurance running, as intensity increases, the active muscle mass becomes progressively more dependent on carbohydrates as energy source, making periodized carbohydrate supplementation crucial for optimal metabolism and energy availability [3,4,5,6,7].

Training and competitive running can induce inflammation during and immediately after endurance performance, impairing optimal recovery processes [8,9]. The pro-inflammatory responses triggered by running initiate a complex cascade of events influencing various immune parameters, including changes in peripheral white blood cell count and, in particular, neutrophils and mononuclear cells and plasma cytokine levels [9,10,11,12]. Since the mid-1990s, many studies have indicated that low carbohydrate supplementation (30–60 g/h) may facilitate a reduction in the inflammatory response to prolonged, intensive running [13,14,15]. Additionally, consuming carbohydrates enhances glucose availability to active muscles, helping to lower nervous system activation, stress hormones, pro-inflammatory signals, and cytokine release from active muscle tissue [16,17].

Repeated muscle contractions, energy deficit, and metabolic stress during endurance running also promote the production of anti-inflammatory mediators, principally interleukin-6 (IL-6), which inhibits pro-inflammatory cytokines like tumor necrosis factor-α (TNF-α), creating an anti-inflammatory environment that counteracts the pro-inflammatory responses associated with high-intensity exercise [18,19,20]. IL-6, produced during the normal physiological response to running, plays a role in modulating the stress response, activating the hypothalamic–pituitary–adrenal axis at multiple levels, resulting in an increased release of cortisol from the adrenal glands. IL-6 also serves a glucoregulatory function, mobilizing and enhancing energy availability during long-distance running when glycogen stores are depleted [21,22]. Moreover, carbohydrate supplementation reduces IL-6 production and hypothalamic–pituitary–adrenal activation, resulting in the moderated release of various hormones, including cortisol [16,23,24,25].

Carbohydrate supplement strategies may also positively impact other post-running inflammatory and stress proteins that increase shortly after the inflammatory response begins, such as C-reactive protein (CRP), a marker of systemic inflammation [9,26,27]. Another example is creatine phosphokinase (CK), a protein involved in muscle metabolism, which serves as a marker of physical stress. CK levels in plasma indicate muscle fiber damage and vary significantly after high-intensity exercise, with eccentric exercise causing more muscle damage than concentric contractions of the same intensity [28].

The metabolic adaptations that occur during long-term physical activity may lead to changes in long-chain ω-3 fatty acid levels. Long-chain ω-3 fatty acids, particularly eicosapentaenoic acid (EPA) and docosahexaenoic acid (DHA) are further molecules pivotal in modulating inflammation. ω-3 fatty acids exert their anti-inflammatory effects through several mechanisms, including the synthesis of anti-inflammatory molecules (mainly resolvins and protectins), the inhibition of pro-inflammatory cytokine production, and the modulation of cell membrane composition, which affects numerous receptor functions and signal transduction pathways. The ω-3 index, which reflects the proportion of EPA and DHA in cell membranes, is an established marker of cardiovascular health and inflammatory status. A higher ω-3 index is associated with reduced inflammation and a lower risk of cardiovascular diseases [29]. Conversely, arachidonic acid (AA) is a precursor for pro-inflammatory eicosanoids, including prostaglandins and leukotrienes, which are potent mediators of inflammation. The AA/EPA ratio is thus a valuable indicator of the balance between pro-inflammatory and anti-inflammatory processes in the body. A higher AA/EPA ratio suggests a dominance of pro-inflammatory processes, whereas a lower ratio indicates a more balanced inflammatory state [30].

The impact of carbohydrate supplementation on inflammation induced by running has been extensively studied. However, previous studies mainly focused on a single type of carbohydrate supplementation, with levels typically ranging from 30 to 60 g/h [8,9,31]. These levels do not meet the carbohydrate intake currently considered optimal during prolonged high-intensity endurance exercise. Moreover, the exercise intensity was typically low to moderate, ranging between 60% and 75% of the minimum speed associated with maximal oxygen consumption (vVO_2max_). The duration of these exercises typically lasted between 90 min and 3 h [13,14,32,33,34]. Additionally, there is no information on how long-distance running with or without carbohydrate supplementation affects short-term lipidomic changes. To this end, this study investigated the effects of a mixed high-carbohydrate supplement (containing a 2:1 ratio of maltodextrin to fructose at a rate of 80 g/h, currently considered the optimal carbohydrate intake) when consumed immediately before, during, and after approximately 1 hour of high-intensity (at 90% vVO_2max_) endurance running on short-term inflammatory markers and lipidomic profiles, with a particular focus on the ω-3 index and AA/EPA ratio.

## 2. Materials and Methods

### 2.1. Study Design and Participants

This study is a randomized placebo-controlled cross-over trial that involves twenty-nine healthy volunteers enrolled from a cohort of long-distance runners in Lombardia, Italy, between June 2023 and September 2023, chosen for the homogeneity of their performance and training. The experimental procedure was conducted from 3 November 2023 to 19 November 2023. Subjects who met the following requirements were eligible for enrollment: (1) healthy male and female subjects; (2) Caucasian ethnicity; (3) medium-to-high-level long-distance runners who can complete 15 km under 65 min; (4) willingness to have samples stored for future research; (5) absence of eating disorders; (6) subjects trained at least three times per week for a minimum of 6 consecutive months. Exclusion criteria included pregnancy and a history of chronic diseases with correlated pharmacological treatments. The study was conducted according to the guidelines laid down in the Declaration of Helsinki and was approved by the institutional review board of the University of Molise (Prot. n. 26/2023—11 October 2023). The protocol complied with the Consolidated Standards of Reporting Trials (CONSORT) guidelines for clinical trials and was registered on the Open Science Framework: https://osf.io/dbqc8 (accessed on 6 August 2024). All participants provided written informed consent.

### 2.2. Randomization and Procedures

The order of interventions in the two separate tests was randomized using RStudio (version 4.3.1). Seven days were allowed between each intervention to ensure that each athlete could return to their baseline state. Non-compliant subjects (e.g., those who developed a cold, fever, or any condition that led to altered inflammatory markers during the observation period) were excluded from the analysis. Weather conditions such as wind, rain, temperature, and humidity were evaluated to ensure the two tests were performed under the same conditions.

**Figure 1:**
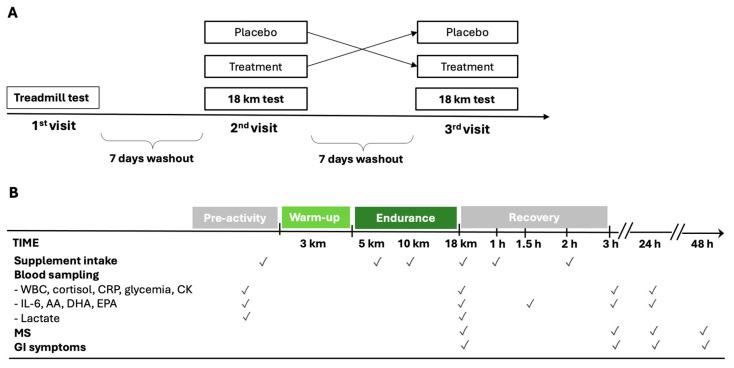
Schematic diagram of cross-over study design (**A**) and experimental protocol during the 2nd and 3rd visits (**B**). AA = arachidonic acid; CK = creatine phosphokinase; CRP = C-reactive protein; DHA = docosahexaenoic acid; MS = muscle soreness; EPA = eicosapentaenoic acid; GI = gastrointestinal; IL-6 = interleukin-6; WBC = white blood cells.

The timing of supplementation, the endurance period, and the sampling schedule of the experimental protocol are shown in Figure 1. During the first evaluation, an incremental ramp test on the treadmill was performed. The gas during exercise and resting were analyzed with a metabolic cart (Quark CPET, Cosmed, Rome, Italy). After a 3 h fast, athletes ran until exhaustion on a treadmill that increased by 0.1 km/h every 12, with a constant inclination of 1%. The starting speed was decided based on the athlete’s level so that the test could last between 8 and 12 min overall. First ventilatory threshold (VT1), second ventilatory threshold (VT2), minimum velocity associated with vVO_2max_, velocity at peak (V_Max_), maximal oxygen consumption (VO_2max_), and respiratory exchange ratio (RER) were measured. Weight and plicometry were also measured before the incremental test. The body fat percentage was determined according to the Jackson and Pollock equation [35,36].

Running athletes were instructed to familiarize themselves with the treadmill in the weeks before the test. Additionally, they were required to abstain from intense physical activity for 48 h before each test. During this period, only rest or low-volume/low-intensity training was permitted, and muscle strength training was strictly prohibited. During the second and third visits, 7 and 14 days after the first visit, the athletes, after an overnight fast, performed the outdoor 18 km endurance test on a flat course (a circuit of 5 km repeated three times with an overall elevation gain of 6 m/loop), composed as follows: 3 km warm-up at an intensity below VT1 and then 15 km at the speed of 90% of vVO_2max_. The weather conditions (temperature, humidity, and wind speed) between the endurance tests were similar.

The measurement of blood lactate from the earlobe (Lactate Pro 2 Arkray, Kyoto, Japan) was performed before the warm-up and at the end of the 15 km run. Alongside these data, heart rate during the test, the rate of perceived exertion (RPE) scale at the end of the test, and weight before and immediately after each test were also collected. Moreover, a 7-point Likert Scale of muscle soreness (MS) was administered to the athletes upon completion of the 15 km and at 3, 24, and 48 h afterward. Any gastrointestinal symptoms (GI) were recorded from the first supplementation up to 24 h after the test.

### 2.3. Supplementation and Dietary Instruction

A total of 60 ml of Carbo Gel C2:1 (Enervit, Milan, Italy) containing 40 g of maltodextrin–fructose 2:1 (composition of 100 g of Carbo Gel C2:1: 44.51 g water, 36.4 g maltodextrin, 18.2 g fructose, 0.26 g xantham gum, 0.42 g citric acid, 0.1 g natural flavoring, 0.1 g potassium sorbate, 3.22 mg niacin, 0.28 mg vitamin B6, and 0.23 mg thiamin) or 60 mL of placebo (composition of 100 g: 98.63 g water, 0.75 g xantham gum, 0.42 g citric acid, 0.1 g natural flavoring, 0.1 g potassium sorbate, 4.26 mg sucralose, 3.22 mg niacin, 0.28 mg vitamin B6, and 0.23 mg thiamin) were administered six times (after the first venous sample immediately before starting the warm-up, at the 5th and 10th km during the 15 km test, immediately after the second venous sample, and after 1 h and 2 h of recovery) with a carbohydrate intake of 80 g/h (Figure 1B). The maltodextrin–fructose 2:1 supplementation and placebo were identical in consistency, acidity, and taste.

All long-distance runners were instructed to follow their usual diet and to maintain the same diet from the day before to the day after each test. However, starting 48 h before the test and continuing until the end of the experimental procedure, athletes were restricted from consuming foods containing significant amounts of ω-3 and ω-6 fatty acids. Additionally, they were instructed not to take ω-3 supplements for 30 days before the test. They were also asked to keep a three-day food diary to assess the caloric and macronutrient intake in their diet. This diary was kept from the day before to the day after the first visit and the test days. The athletes arrived in the morning in a fasted state before each 15 km test.

### 2.4. Glycemia, Inflammatory, and Damage Biomarkers

Blood samples were taken immediately before and after the 15 km run and 3 h and 24 h from the end of each test. Before each blood sample, the athlete remained seated for at least 5 min. During the 3 h recovery, the athletes mostly stayed seated, and only short walks were allowed in the laboratory room. Venipunctures were performed for each blood sample. From the venous blood samples, the following parameters were analyzed: glycemia, complete blood count, IL-6, CRP, cortisol, and CK. An additional blood sample after 1.5 h from the end of each test was taken specifically for IL-6 analysis.

### 2.5. Lipidomic Analysis

Whole blood fatty acid composition on dried blood spots (before and after the 15 km run, after 1.5 h, 3 h, and 24 h from the end of each test) was analyzed by an external service at the University of Milan using high-resolution capillary gas chromatography, as previously described [37].

### 2.6. Statistical Analysis

Descriptive characteristics were reported as frequency and percentage for categorical data, mean and standard deviation (SD) or median, and I–III quartile for continuous variables, where appropriate. Comparison between baseline characteristics was performed through the Chi-squared test, Fisher’s exact test, Mann–Whitney U test, or t-test depending on the nature of the variable. The trend of each variable between the two arms (maltodextrin–fructose 2:1 supplementation or placebo) was shown through a plot where the x-axis represents the time (x = 0 was immediately before, 1 was post-running, 1.5 was the measure after 1 h and a half, and 3 was the measures take 3 h and 24 h post-running), and the y-axis represents the values for each parameter of interest. The hypothesis of a normal distribution at all times for each variable was evaluated for each variable via the Shapiro–Wilk test.

Two-way repeated ANOVA was performed to compare the effect across the different measurement times and between the two interventions on the outcomes. To model the within-subjects variables, a subject term was also included. Then, linear mixed-effects models were performed including treatment, time, and the interaction term between the two arms, with a random intercept for each participant. The same models were used to compare the effect of the interventions on the other interesting variables. Analysis from both approaches produces consistent results. Since the CRP values were not normally distributed, these values were categorized as ≤0.16 mg/dL or >0.16 mg/dL. A value of 0.16 mg/dL defines the minimum detectable level, and a high level of CRP indicates a more inflammatory status. Then, GEE models (generalized estimating equations) were fitted to evaluate the effect of time and the effect of treatment. All models were adjusted for the intervention sequence. The linear regression model was used to evaluate the association of the AA/EPA ratio at baseline on CK variation (from baseline to 24 h post-running). The AA/EPA ratio was categorized as <20, 20–30, and >30. The coefficient (β) and its 95% confidence interval (CI 95%) were provided.

All analyses were performed using RStudio (version 4.3.1). A two-tailed *p*-value of 0.05 was considered significant. Since some analyses involved multiple tests, the *p*-values were adjusted with a Bonferroni correction.

## 3. Results

### 3.1. Study Population

From the 43 enrolled participants, 29 medium-to-high-level runners were randomized, and 26 (4 females and 22 males) completed the two sequences and were included in the analysis (Figure S1). Individual characteristics of the participants are presented in Table S1. The median age was 32 years (I–III quartiles = 24.3–40) and the mean VO_2max_ equal to 61.84 ± 5.33 mL/kg·min. No significant differences were found between included (n = 26) and excluded (n = 3) participants (Table S1). The endurance performances for the two arms, considering the cross-over design, are presented in Table 1. No significant differences were found in the 15 km endurance test results between the two arms. Additionally, only a few athletes presented GI symptoms, without any differences.

Baseline blood markers (both inflammatory and lipidomic) were not significantly different between placebo and treatment arms (Table 2). 

### 3.2. Glycemia

**Figure 2:**
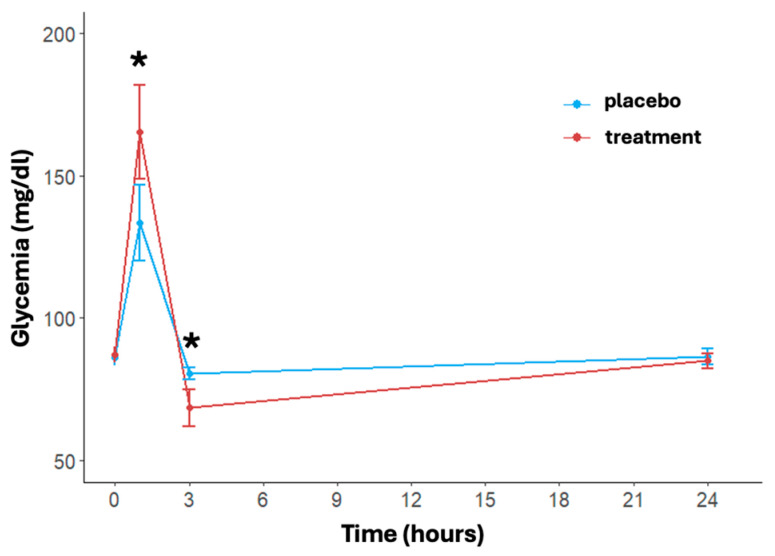
Effects of treatment and placebo over time (at baseline, post-running, after 3 and 24 h post-running) on blood glucose levels. Data are reported as mean ± standard error of the mean. * significant difference between treatments.

The trend of blood glucose levels over time and between treatments is shown in Figure 2. A significant effect of time (*p* < 0.001) in both treatment and placebo arms was found. In detail, immediately post-activity, the glycemia showed a notable increase in both arms with a faster and significant increase in the treatment group (placebo: 133.46 ± 34.35 mg/dL, treatment: 165.42 ± 42.85 mg/dL, *p* = 0.004). At three hours post-running, glucose levels dropped significantly more in the treatment group (placebo: 80.50 ± 5.58 mg/dL, treatment: 68.58 ± 16.81 mg/dL, *p* = 0.002). At 24 h, blood glucose levels in both arms returned close to the baseline value, with no significant differences between the two arms (placebo: 86.58 ± 7.21 mg/dL, treatment: 84.96 ± 6.77 mg/dL, *p* = 0.432). The delta differences between post-activity and 3 h measurements compared to 24 h levels were significant for both arms (*p* < 0.001).

### 3.3. Inflammatory Biomarkers

**Figure 3:**
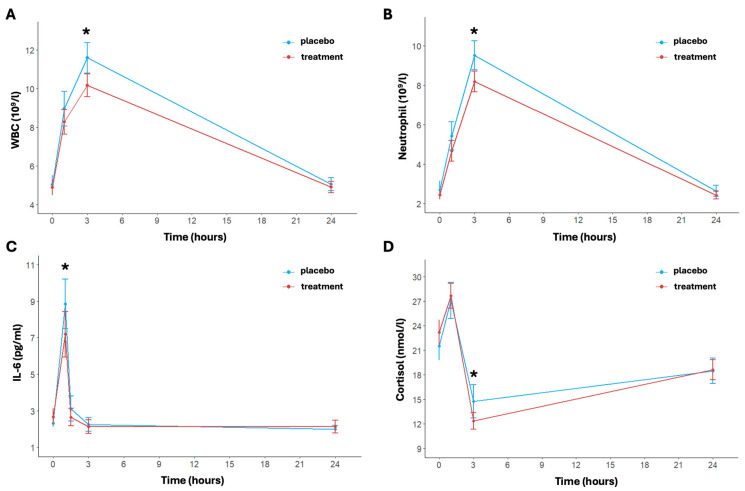
Inflammatory indices pre- and post-activity and at 3 and 24 h post-activity: WBC (**A**), neutrophil (**B**), IL-6 (**C**), and cortisol (**D**). Data are reported as mean ± standard error of the mean. * significant difference between the two arms.

The inflammatory response over time and between the two arms is shown in Figure 3. In particular, regarding the white blood cell count, the model revealed significant effects of time (*p* < 0.001). Specifically, the time effect was always significant except for the delta between baseline and 24 h post-running; this trend was shown in both arms (Figure 3A). The white blood cell concentration in the placebo group increased from a baseline of 5.04 ± 1.47 × 10^9^/L to 11.59 ± 2.43 × 10^9^/L at 3 h then decreased to 5.05 ± 1.04 × 10^9^/L at 24 h. Instead, the treatment group showed a more moderate increase, with levels rising from 4.88 ± 1.25 × 10^9^/L at baseline to 10.16 ± 1.82 × 10^9^/L at 3 h post-running and returning to 4.90 ± 0.93 × 10^9^/L at 24 h. The treatment effect was notably significant at 3 h post-running (*p* < 0.001); also, the change in the delta between baseline and 3 h post-running differed significantly between the two arms (*p* = 0.0137).

The concentration of neutrophils showed a significant transient increase immediately after exercise, peaking at 3 h post-running for both arms (*p* < 0.001) (Figure 3B). In particular, under placebo conditions, neutrophil levels increased from a baseline mean of 2.71 ± 1.39 × 10^9^/L to 9.51 ± 2.29 × 10^9^/L at 3 h post-running (*p* < 0.001). In the treatment group, the increase was notably attenuated, rising from a baseline of 2.45 ± 0.74 × 10^9^/L to 8.18 ± 1.63 × 10^9^/L at 3 h post-running. The difference in concentration of neutrophil between the two arms was statistically significant at 3 h post-running and in the change in delta between baseline and 3 h post-running (*p* = 0.018 and *p* = 0.009, respectively). Finally, as to the white blood cell count trend, the time effect was not significant for the delta between baseline and 24 h post-running in both arms. 

The levels of IL-6 changed significantly over time (*p* < 0.001); in particular, IL-6 levels were significantly elevated immediately post-running then returned to baseline values 24 h post-exercise (Figure 3C). The placebo group showed a baseline IL-6 level of 2.31 ± 0.66 pg/mL, which significantly increased to 8.84 ± 4.22 pg/mL immediately post-running and a rapid decrease at 1.5 h post-running (3.12 ± 2.15 pg/mL) (*p* < 0.001 for time). In the treatment group, at baseline, IL-6 was equal to 2.66 ± 1.45 pg/mL, rising to 7.19 ± 3.88 pg/mL post-running and returning to basal levels of 2.65 ± 1.51 pg/mL at 1.5 h post-running (*p* < 0.001 for time). A significant difference between placebo and treatment was found after exercise (*p* < 0.049) and from pre- and immediate post-running (*p* = 0.007).

Lastly, a significant increase in cortisol concentration levels was also observed over time in both arms (*p* < 0.001) (Figure 3D). In particular, the placebo group’s baseline cortisol concentration was 21.52 ± 5.50 nmol/L, which increased to 27.08 ± 6.92 nmol/L post-running (*p* < 0.001). For the treatment group, baseline levels were similar to the placebo group and equal to 23.19 ± 4.71 nmol/L, increasing to 27.65 ± 4.79 nmol/L post-running (*p* < 0.001). Then, after 3 h post-running, cortisol levels in the placebo group had significantly decreased to 14.74 ± 6.30 nmol/L (*p* < 0.001). This decrease is increasingly marked in the treatment group (12.35 ± 3.20 nmol/L, *p* < 0.001), indicating a more rapid recovery (*p* = 0.046 for treatment effect). The cortisol increased at 24 h in both arms, reaching similar levels (placebo: 18.47 ± 4.81 nmol/L; treatment: 18.60 ± 3.81 nmol/L, *p* = 0.916).

**Figure 4:**
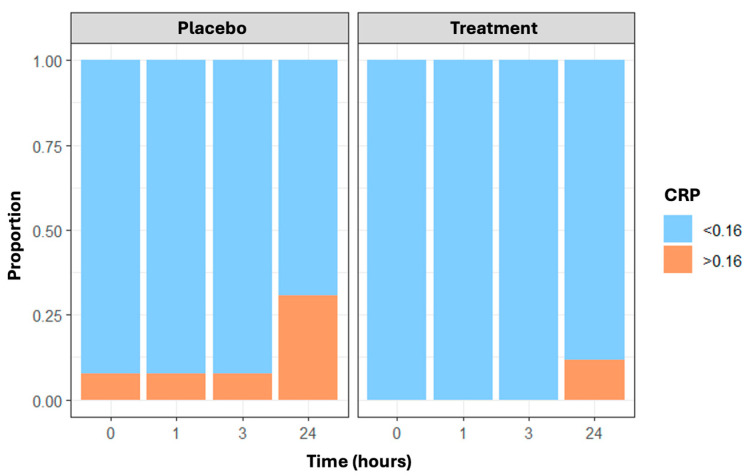
The proportion of CRP values not identifiable (≤0.16 mg/dL vs. >0.16 mg/dL) in placebo and treatment arms over time (at baseline, post-running, and 3 and 24 h post-running).

Finally, CRP values greater than 0.16 mg/dL—the cut-off indicating the minimum detectable level—changed over time and were more frequent in the placebo group than in the treatment group, with the maximum difference at 24 h (n = 8 and 3 subjects with CRP less than 0.16 mg/dL in the placebo and treatment arms, respectively) (Figure 4). Results are confirmed through the generalized estimating equations (GEE) model, which showed a statistically significant time effect (*p* = 0.013) and treatment effect (*p* = 0.006).

### 3.4. Lipidomic Profile, ω-3 Index and AA/EPA Ratio

Regarding the fatty acid markers, a significant time effect was found in the treatment and placebo arms for both AA and EPA, along with a significant variation over time only for the treatment in DHA (Table 3). In particular, AA shows that at baseline, both the placebo and treatment groups had similar levels (8.32 ± 1.80% and 8.40 ± 1.51%, respectively). Post-running, the AA levels increased slightly in both groups, with the placebo group maintaining higher levels (8.55 ± 1.67%) compared to the treatment group (8.45 ± 1.07%). After 1.5 h, the AA levels remained stable in the treatment group (8.69 ± 1.18%), while slightly increasing in the placebo group (8.62 ± 1.54%). At the 3 h mark, the AA levels peaked in both groups, with the placebo group at 9.08 ± 1.54% and the treatment group at 8.95 ± 1.43%. After 24 h, the AA levels decreased in both groups but were significantly higher (*p* < 0.001) in the placebo group (8.45 ± 1.69%) compared to the treatment group (8.10 ± 1.22%). Significant differences over time were noted in the placebo group at all time points and in the treatment group at most time points, with a notable significant difference between the groups observed after 24 h. EPA levels showed significant time effects, with values in the placebo group increasing from 0.34 ± 0.13% to 0.37 ± 0.11% at 3 h then reducing to 0.33 ± 0.11% at 24 h post-running. The treatment group exhibited an increase from 0.38 ± 0.14% to 0.40 ± 0.15% at 3 h, followed by a decrease to 0.35 ± 0.12% at 24 h. DHA levels similarly increased and then decreased in both arms. However, the effect of time is significant only in the treatment group. In particular, rising from 2.04 ± 0.80% to 2.12 ± 0.66% at 3 h and reducing to 1.91 ± 0.75% at 24 h post-running in the placebo group. The treatment group’s DHA levels significantly increased from 1.90 ± 0.64% (baseline) to 2.11 ± 0.73% at 3 h then significantly decreased to 1.79 ± 0.59% at 24 h. The *p*-values of the interaction terms (treatment × time) are shown in Table S2. Moreover, there is no significant effect of either time or treatment on total saturated fatty acids, stearic acid, and palmitic acid (Figure S2).

**Figure 5:**
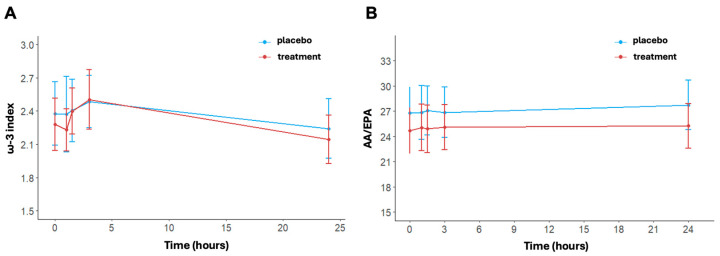
ω-3 index (**A**) and AA/EPA ratio (**B**) at baseline, post-activity, after 1.5, 3, and 24 h post-running. Data are reported as mean ± standard error of the mean.

The ω-3 index changed significantly over time in the treatment arm (*p* < 0.001); (Figure 5A). No significant time effect was shown for the placebo (*p* = 0.516). In detail, the values of ω-3 at baseline were 2.38 ± 0.89% in the placebo group and 2.28 ± 0.73% in the treatment group. Post-running, there was an increase in the ω-3 index in both arms, reaching values of 2.49 ± 0.73% in the placebo group and 2.50 ± 0.84% in the treatment group at 3 h. At 24 h post-running, ω-3 index levels decreased with respect to levels at 3 h post-running in both arms but did so significantly only in the treatment group (*p* < 0.001). No differences between arms were found at each timepoint (at 24 h, placebo: 2.24 ± 0.84% vs. treatment: 2.14 ± 0.68%, *p* = 0.579).

Regarding the AA/EPA ratio, no statistically significant variation was found over time (Figure 5B). In detail, at baseline, the AA/EPA ratio was equal to 26.8 ± 9.5 in the placebo group and 24.68 ± 8.45 in the treatment group. The AA/EPA ratio was similar in the placebo group immediately post-running (26.84 ± 10.03) and at 3 h post-running (26.86 ± 9.36). In contrast, the treatment group exhibited a small increase, with an AA/EPA ratio of 25.05 ± 8.63 immediately post-running and 25.09 ± 8.29 at 3 h post-running. AA/EPA is higher in the placebo group compared to the treatment group at 24 h post-running. Indeed, the AA/EPA ratio had decreased to near baseline levels in the treatment group (25.26 ± 8.25), while the AA/EPA ratio remained increased in the placebo group (27.74 ± 25.26). The *p*-values of the interaction terms (treatment x time) are shown in Table S2.

### 3.5. Creatine Phosphokinase (CK)

The results showed a significant effect of time on CK levels for both the placebo arm (*p* = 0.001) and the treatment arm (*p* = 0.005). Specifically, the changes from baseline to the three time points after exercise (e.g., immediately post-running, after 3 h, and 24 h) were significantly different for both arms. In particular, the CK values for the placebo group were at baseline 259.92 ± 321.55 IU/L, immediately post-running 360.96 ± 377.80 IU/L (*p* < 0.001), 3 h post-running 348.12 ± 352.36 IU/L (*p* < 0.001), and 24 h post-running 410.77 ± 486.56 IU/L (*p =* 0.002). For the treatment group, the values were at baseline 207.62 ± 111.37 IU/L, immediately post-running 294.77 ± 153.09 IU/L (*p* < 0.001), 3 h post-running 287.27 ± 139.04 IU/L (*p* < 0.001), and 24 h post-running 316.73 ± 198.14 IU/L (*p =* 0.009). The differences in CK values between the two arms showed that the CK levels were generally lower but not statistically different in the treatment group compared to the placebo group.

**Figure 6:**
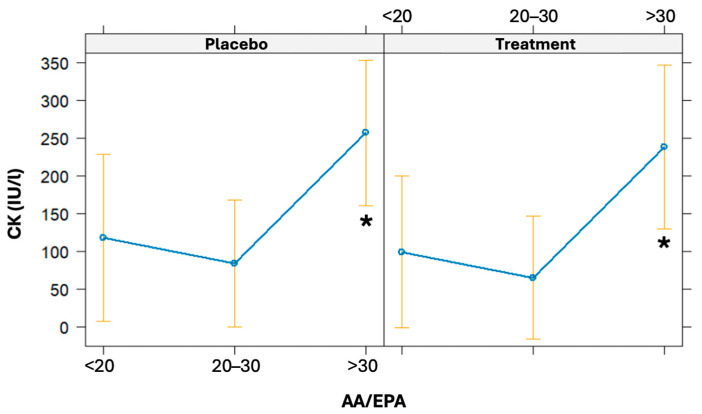
Effects plot of the linear regression models used to predict CK values given a baseline AA/EPA ratio. The models were also adjusted for the treatment arm. * significant difference.

The linear regression model was used to evaluate the association of the AA/EPA ratio at baseline on CK variation (from baseline to 24 h post-running). The AA/EPA ratio was categorized as <20, 20–30, and >30. Even though it was not statistically significant, there is a borderline association observed between baseline AA/EPA ratios and increments in CK levels (β = 5.16, CI 95% = [−0.39; 10.71], *p* = 0.068). High baseline values of the AA/EPA ratio (particularly if >30) are associated with a statistically significant increase in CK 24 h post-running (*p* = 0.031) (Figure 6).

## 4. Discussion

This study shows that supplementation with a high dosage of 2:1 maltodextrin–fructose formulation to medium-to-high-level athletes before, during, and after a 15 km run at 90% vVO_2max_ intensity may mitigate acute post-activity inflammatory stress, with a concomitant reduction in white blood cells, particularly neutrophils, IL-6, cortisol, and CRP levels. Previous studies that investigated the association of carbohydrate supplementation during exercise on inflammation have shown conflicting results and critical limitations mainly attributable to the following aspects: inadequate carbohydrate supplementation (less than 60 g/h, the maximum glucose oxidation rate) and athletes involved in exercise at intensities where carbohydrates were not the only energy source [13,14,15,26,31,38]. In our study, the athletes ran at an intensity where carbohydrates were used exclusively as an energy source, as evidenced by the mean lactate values at the end of the test, and they were supplemented with the carbohydrate intake currently considered optimal during prolonged high-intensity endurance exercise (80 g/h) [39]. Carbohydrates from a single source, such as glucose, can only be oxidized at approximately 60 g/h because there is a limitation in the intestinal absorption rate of a single type of carbohydrate due to transporter saturation [40]. The ingestion of carbohydrates that use different transporters may increase total carbohydrate absorption and their oxidation efficiency, allowing for the optimal carbohydrate intake during high-intensity endurance exercise. Indeed, when a combination of carbohydrates is ingested (e.g., glucose and fructose, maltodextrins, and fructose, or glucose, sucrose, and fructose), it is possible to achieve oxidation rates of more than 100 g/h, avoiding potential gastrointestinal complications [41,42,43,44,45,46,47]. Regarding the glycemic levels during the test, it is important to note that both arms exhibited a peak in glycemic levels after exercise, with the treatment arm showing a significantly more pronounced increase. This increase in glycemic levels in both arms may be attributed to the strong adrenergic activation induced by the endurance test [48]. The peak in glycemic levels rapidly decreased in both arms 3 h post-running, with a more significant reduction observed in the treatment group, avoiding hypoglycemia.

Our main findings suggest that carbohydrate supplementation may mitigate the short-term inflammatory and stress response induced by endurance running. Indeed, following endurance running, which involves repetitive eccentric muscle contraction and the depletion of muscle glycogen stores, it is known that there is a transient increase in the pro-inflammatory state accompanied by the mobilization of white blood cells, particularly neutrophils, and an increase in CRP, as confirmed by our data [9,10,11,12]. The effect of supplementation with a high dose of 2:1 maltodextrin and fructose ensures a significant reduction in these inflammatory parameters compared to the placebo within 24 h after the end of exercise. In parallel, there is the activation of compensatory mechanisms, where IL-6 may play a crucial role. It is acutely released from active muscle fibers in response to increased exercise duration, intensity, and muscle glycogen depletion. IL-6 promotes communication between muscles and other organs, aiding in the coordinated response necessary to maintain muscle energy homeostasis and anti-inflammatory behavior [20]. Optimal supplementation with high doses of a maltodextrin–fructose supplement before, during, and after exercise maintains energy availability, particularly carbohydrate availability, during high-intensity endurance performance, reducing metabolic stress and subsequently lowering anti-inflammatory mechanisms such as the IL-6 release [21,22]. The reduction in metabolic stress may be linked to the direct or indirect (IL-6-mediated) effects of maltodextrin–fructose on the hypothalamic–pituitary–adrenal axis, leading to a decrease in cortisol levels, a hormone also known for its hyperglycemic activity [16,23,24,25].

The endogenous response of ω-3 and ω-6 fatty acids in inflammatory processes in running has been inadequately explored [49]. Moreover, there is no information regarding their role in transient inflammatory mechanisms after endurance exercise. It is well established that AA-derived eicosanoids promote inflammation and other physiological processes, while EPA and DHA-derived mediators have potent anti-inflammatory properties and contribute to resolving inflammation [29,50]. In both arms, an increase in AA levels is observed, reaching its peak at 3 h post-running, likely as an inflammatory response induced by high-intensity endurance running. However, it is noteworthy that 24 h post-running, the reduction in these levels, which occurs in both arms, is marked in the treatment arm compared to the placebo, reaching values lower than baseline. A similar trend is observed for DHA in both arms, although the effect of time is significant only in the treatment group. Regarding EPA, there is a more rapid increase in the treatment arm at 1.5 h post-running, which is significant compared to the placebo, but this significance is lost after 3 h. Overall, these fluctuations in polyunsaturated fatty acids appear to be associated with high-intensity endurance activity, but are more pronounced in the treatment arm, suggesting a specific mechanism related to this type of fatty acids. Supporting this observation, the lack of variation in saturated fatty acid levels indicates that the changes in polyunsaturated fatty acid levels are not the expression of their possible use as energy substrates, with a possible specific contribution to inflammatory regulation. Equally interesting is the AA/EPA ratio trend, which remains consistently higher in the placebo group than in the treated group 24 h post-test. In the treated group, the ratio values normalized, returning to baseline levels. This supports the idea that in the absence of carbohydrate supplementation, a pro-inflammatory state persists for a longer duration in athletes. While acute supplements did not directly influence post-running CK levels, the high pre-activity AA/EPA values were associated with higher muscle damage post-activity, as indicated by elevated CK levels at 24 h, particularly in athletes with a very high AA/EPA ratio (>30).

Although the strengths of this study listed so far are numerous, the main limitations include (1) the small number of runners involved; (2) the limited number of women enrolled in the study population, without complete information about the phase of their menstrual cycle [51]; and (3) the narrow range of inflammatory cytokines analyzed, which restricts a more comprehensive evaluation of this type of supplementation. Future research is needed to elucidate fully the mechanisms behind carbohydrate supplementation and its benefits on running training, performance, and recovery.

## 5. Conclusions

Supplementation with a high-carbohydrate mixture of maltodextrin–fructose at a 2:1 ratio can acutely mitigate inflammation during a one-hour endurance activity of moderate-to-high intensity. Additionally, at 24 h after the endurance activity, this supplementation may significantly reduce the post-endurance increase in AA levels. These effects could be beneficial for athletes engaging in frequent, high-intensity activities. However, dedicated prospective studies are needed to verify this hypothesis.

## Figures and Tables

**Table 1:** Endurance performances of placebo and treatment arms.

	Placebo	Treatment	*p*-Value *
3 km test velocity (km/h)	13.31 ± 0.75	13.44 ± 0.71	0.509
15 km test velocity (km/h)	15.59 ± 1.46	15.59 ± 1.45	0.992
Mean heart rate during 3 km (bpm)	145.96 ± 10.1	146.64 ± 11.96	0.829
Mean heart rate during 15 km (bpm)	171.38 ± 8.85	171.23 ± 9.18	0.951
Maximal heart rate during 15 km (bpm)	179.58 ± 9.70	179.46 ± 8.56	0.964
Rating of perceived exertion	7.15 ± 1.74	6.96 ± 1.66	0.685
Baseline lactate (mmol/L)	1.35 ± 0.21	1.32 ± 0.24	0.629
Post-running lactate (mmol/L)	5.14 ± 1.95	4.64 ± 1.44	0.298
Post–pre test delta weight (kg)	−1.33 ± 0.43	−1.28 ± 0.40	0.690
GI symptoms (n)	6 (23.1)	4 (15.4)	0.481

bpm = beats per minute; * *p*-value refers to the comparison between the two arms. Results were shown as mean ± SD or frequency and percentage (%). GI = gastronitestinal.

**Table 2:** Baseline characteristics of inflammatory and damage markers and lipidomic profile.

	Overall	Placebo	Treatment	*p*-Value *
Inflammatory and damage markers at baseline				
White blood cells (n × 10^9^)	4.96 ± 1.35	5.04 ± 1.47	4.88 ± 1.25	0.671
Neutrophil (n × 10^9^)	2.58 ± 1.11	2.71 ± 1.39	2.45 ± 0.74	0.409
Cortisol (nmol/L)	22.36 ± 5.14	21.52 ± 5.50	23.19 ± 4.71	0.247
IL-6 (pg/mL)	2.49 ± 1.13	2.31 ± 0.66	2.66 ± 1.45	0.269
CRP (mg/dL)	0.16 ± 0.18	0.19 ± 0.26	0.14 ± 0.03	0.333
CK (IU/L)	233.77 ± 239.71	259.92 ± 321.55	207.62 ± 111.37	0.437
Lipidomic profile at baseline				
ω-3 index (%)	2.33 ± 0.81	2.38 ± 0.89	2.28 ± 0.73	0.669
EPA (%)	0.36 ± 0.13	0.34 ± 0.13	0.38 ± 0.14	0.329
DHA (%)	1.97 ± 0.72	2.04 ± 0.80	1.90 ± 0.64	0.509
AA (%)	8.36 ± 1.64	8.32 ± 1.80	8.40 ± 1.51	0.878
AA/EPA ratio	25.74 ± 8.96	26.80 ± 9.50	24.68 ± 8.45	0.399

* *p*-value refers to the comparison between the two arms. Results were shown as mean ± SD or percentage (%). AA = arachidonic acid; CK = creatine phosphokinase; CRP = C-protein reactive; DHA = docosahexaenoic acid; EPA = eicosapentaenoic acid; IL-6 = interleukin 6; IU = international units.

**Table 3:** Distribution of AA, DHA, and EPA over time between the two arms (placebo and treatment).

	AA (%)		DHA (%)		EPA (%)	
Timepoints	Placebo	Treatment	Placebo	Treatment	Placebo	Treatment
Baseline	8.32 ± 1.80 ^#^	8.40 ± 1.51 ^#^	2.04 ± 0.80	1.90 ± 0.64 ^#^	0.34 ± 0.13 ^#^	0.37 ± 0.14
Post-running	8.55 ± 1.67 ^#^	8.45 ± 1.07	2.02 ± 1.00	1.85 ± 0.50 ^#^	0.34 ± 0.12	0.37 ± 0.14
After 1.5 h	8.62 ± 1.54	8.69 ± 1.18 ^#^	2.06 ± 0.82	2.01 ± 0.57 ^#^	0.34 ± 0.11 *	0.38 ± 0.14 *^,#^
After 3 h	9.08 ± 1.54 ^#^	8.95 ± 1.43 ^#^	2.12 ± 0.66	2.11 ± 0.73 ^#^	0.36 ± 0.12 ^#^	0.39 ± 0.15 ^#^
After 24 h	8.45 ± 1.69 *	8.10 ± 1.22 *^,#^	1.91 ± 0.75	1.79 ± 0.59 ^#^	0.32 ± 0.12 ^#^	0.35 ± 0.12 ^#^

AA = arachidonic acid; DHA = docosahexaenoic acid; EPA = eicosapentaenoic acid. * significant difference between the two arms; ^#^ significant difference over time.

## Data Availability

The data presented in this study are available on request from the corresponding author due to reasonable request. The data are not publicly available due to privacy.

## Supplementary Materials

The following supporting information can be downloaded at https://www.mdpi.com/article/10.3390/nu16183078/s1: Table S1: Baseline characteristics of the enrolled athletes. Table S2: Descriptive statistics of interaction effects for all lipidomic parameters. Figure S1: Consort flow diagram depicting the flow of participants through the cross-over trial. Figure S2: total saturated fatty acids (**A**) palmitic acid (**B**) and stearic acid (**C**) at baseline, post-activity, after 1.5, 3, and 24 h post-running.

## References

[1] Amawi A., AlKasasbeh W., Jaradat M., Almasri A., Alobaidi S., Hammad A.A., Bishtawi T., Fataftah B., Turk N., Al Saoud H. (2023). Athletes’ nutritional demands: A narrative review of nutritional requirements. Front. Nutr..

[2] Martín-Rodríguez A., Belinchón-deMiguel P., Rubio-Zarapuz A., Tornero-Aguilera J.F., Martínez-Guardado I., Villanueva-Tobaldo C.V., Clemente-Suárez V.J. (2024). Advances in Understanding the Interplay between Dietary Practices, Body Composition, and Sports Performance in Athletes. Nutrients.

[3] Jeukendrup A. (2014). A step towards personalized sports nutrition: Carbohydrate intake during exercise. Sports Med..

[4] Madsen K., Pedersen P.K., Rose P., Richter E.A. (1990). Carbohydrate supercompensation and muscle glycogen utilization during exhaustive running in highly trained athletes. Eur. J. Appl. Physiol. Occup. Physiol..

[5] Sherman W.M., Costill D.L., Fink W.J., Miller J.M. (1981). Effect of exercise-diet manipulation on muscle glycogen and its subsequent utilization during performance. Int. J. Sports Med..

[6] Gonzalez J.T., Fuchs C.J., Betts J.A., van Loon L.J.C. (2016). Liver glycogen metabolism during and after prolonged endurance-type exercise. Am. J. Physiol. Endocrinol. Metab..

[7] Jentjens R., Jeukendrup A.E. (2003). Determinants of post-exercise glycogen synthesis during short-term recovery. Sports Med..

[8] Gleeson M., Bishop N.C., Stensel D.J., Lindley M.R., Mastana S.S., Nimmo M.A. (2011). The anti-inflammatory effects of exercise: Mechanisms and implications for the prevention and treatment of disease. Nat. Rev. Immunol..

[9] Allen J., Sun Y., Woods J.A. (2015). Exercise and the Regulation of Inflammatory Responses. Prog. Mol. Biol. Transl. Sci..

[10] Peake J.M., Neubauer O., Walsh N.P., Simpson R.J. (2017). Recovery of the immune system after exercise. J. Appl. Physiol..

[11] Hennigar S.R., McClung J.P., Pasiakos S.M. (2017). Nutritional interventions and the IL-6 response to exercise. FASEB J..

[12] Silveira L.S., de Moura Mello Antunes B., Luis Araujo Minari A., Vagner Thomatieli Dos Santos R., Rosa Neto J.C., Santos Lira F. (2016). Macrophage Polarization: Implications on Metabolic Diseases and the Role of Exercise. Crit. Rev. Eukaryot. Gene Expr..

[13] Nehlsen-Cannarella S.L., Fagoaga O.R., Nieman D.C., Henson D.A., Butterworth D.E., Schmitt R.L., Bailey E.M., Warren B.J., Utter A., Davis J.M. (1997). Carbohydrate and the cytokine response to 2.5 h of running. J. Appl. Physiol..

[14] Nieman D.C., Davis J.M., Henson D.A., Walberg-Rankin J., Shute M., Dumke C.L., Utter A.C., Vinci D.M., Carson J.A., Brown A. (2003). Carbohydrate ingestion influences skeletal muscle cytokine mRNA and plasma cytokine levels after a 3-h run. J. Appl. Physiol..

[15] Davison G., Gleeson M. (2005). Influence of acute vitamin C and/or carbohydrate ingestion on hormonal, cytokine, and immune responses to prolonged exercise. Int. J. Sport Nutr. Exerc. Metab..

[16] Nieman D.C., Mitmesser S.H. (2017). Potential Impact of Nutrition on Immune System Recovery from Heavy Exertion: A Metabolomics Perspective. Nutrients.

[17] Bermon S., Castell L., Calder P., Bishop N., Blomstrand E., Mooren F., Krüger K., Kavazis A., Quindry J., Senchina D. (2017). Consensus Statement Immunonutrition and Exercise. Exerc. Immunol. Rev..

[18] Pedersen B.K., Febbraio M.A. (2008). Muscle as an endocrine organ: Focus on muscle-derived interleukin-6. Physiol. Rev..

[19] Kistner T.M., Pedersen B.K., Lieberman D.E. (2022). Interleukin 6 as an energy allocator in muscle tissue. Nat. Metab..

[20] Nash D., Hughes M.G., Butcher L., Aicheler R., Smith P., Cullen T., Webb R. (2023). IL-6 signaling in acute exercise and chronic training: Potential consequences for health and athletic performance. Scand. J. Med. Sci. Sports.

[21] Steensberg A., Febbraio M.A., Osada T., Schjerling P., Van Hall G., Saltin B., Pedersen B.K. (2001). Interleukin-6 production in contracting human skeletal muscle is influenced by pre-exercise muscle glycogen content. J. Physiol..

[22] Petersen A.M.W., Pedersen B.K. (2005). The anti-inflammatory effect of exercise. J. Appl. Physiol..

[23] Bartlett J.D., Hawley J.A., Morton J.P. (2015). Carbohydrate availability and exercise training adaptation: Too much of a good thing?. Eur. J. Sport Sci..

[24] Hawley J.A., Morton J.P. (2014). Ramping up the signal: Promoting endurance training adaptation in skeletal muscle by nutritional manipulation. Clin. Exp. Pharmacol. Physiol..

[25] Naderi A., Gobbi N., Ali A., Berjisian E., Hamidvand A., Forbes S.C., Koozehchian M.S., Karayigit R., Saunders B. (2023). Carbohydrates and Endurance Exercise: A Narrative Review of a Food First Approach. Nutrients.

[26] Liang Y., Chen Y., Yang F., Jensen J., Gao R., Yi L., Qiu J. (2022). Effects of carbohydrate and protein supplement strategies on endurance capacity and muscle damage of endurance runners: A double blind, controlled crossover trial. J. Int. Soc. Sports Nutr..

[27] Fedewa M.V., Hathaway E.D., Ward-Ritacco C.L. (2017). Effect of exercise training on C reactive protein: A systematic review and meta-analysis of randomised and non-randomised controlled trials. Br. J. Sports Med..

[28] Cerqueira É., Marinho D.A., Neiva H.P., Lourenço O. (2020). Inflammatory Effects of High and Moderate Intensity Exercise—A Systematic Review. Front. Physiol..

[29] Medoro A., Buonsenso A., Centorbi M., Calcagno G., Scapagnini G., Fiorilli G., Davinelli S. (2024). Omega-3 Index as a Sport Biomarker: Implications for Cardiovascular Health, Injury Prevention, and Athletic Performance. J. Funct. Morphol. Kinesiol..

[30] Davinelli S., Intrieri M., Corbi G., Scapagnini G. (2021). Metabolic indices of polyunsaturated fatty acids: Current evidence, research controversies, and clinical utility. Crit. Rev. Food Sci. Nutr..

[31] Nieman D.C. (1998). Influence of carbohydrate on the immune response to intensive, prolonged exercise. Exerc. Immunol. Rev..

[32] Robson-Ansley P., Walshe I., Ward D. (2011). The effect of carbohydrate ingestion on plasma interleukin-6, hepcidin and iron concentrations following prolonged exercise. Cytokine.

[33] Sim M., Dawson B., Landers G., Wiegerinck E.T., Swinkels D.W., Townsend M.A., Trinder D., Peeling P. (2012). The effects of carbohydrate ingestion during endurance running on post-exercise inflammation and hepcidin levels. Eur. J. Appl. Physiol..

[34] Ihalainen J.K., Vuorimaa T., Puurtinen R., Hämäläinen I., Mero A.A. (2014). Effects of carbohydrate ingestion on acute leukocyte, cortisol, and interleukin-6 response in high-intensity long-distance running. J. Strength Cond. Res..

[35] Jackson A.S., Pollock M.L. (1978). Generalized equations for predicting body density of men. Br. J. Nutr..

[36] Jackson A.S., Pollock M.L., Ward A. (1980). Generalized equations for predicting body density of women. Med. Sci. Sports Exerc..

[37] Cadario F., Pozzi E., Rizzollo S., Stracuzzi M., Beux S., Giorgis A., Carrera D., Fullin F., Riso S., Rizzo A.M. (2019). Vitamin D and ω-3 Supplementations in Mediterranean Diet During the 1st Year of Overt Type 1 Diabetes: A Cohort Study. Nutrients.

[38] Nieman D.C., Henson D.A., Gojanovich G., Davis J.M., Murphy E.A., Mayer E.P., Pearce S., Dumke C.L., Utter A.C., McAnulty S.R. (2006). Influence of carbohydrate on immune function following 2 h cycling. Res. Sports Med..

[39] San-Millán I., Brooks G.A. (2018). Assessment of Metabolic Flexibility by Means of Measuring Blood Lactate, Fat, and Carbohydrate Oxidation Responses to Exercise in Professional Endurance Athletes and Less-Fit Individuals. Sports Med..

[40] Jeukendrup A.E. (2004). Carbohydrate intake during exercise and performance. Nutrition.

[41] Jentjens R.L.P.G., Venables M.C., Jeukendrup A.E. (2004). Oxidation of exogenous glucose, sucrose, and maltose during prolonged cycling exercise. J. Appl. Physiol..

[42] Jentjens R.L.P.G., Moseley L., Waring R.H., Harding L.K., Jeukendrup A.E. (2004). Oxidation of combined ingestion of glucose and fructose during exercise. J. Appl. Physiol..

[43] Jentjens R.L.P.G., Shaw C., Birtles T., Waring R.H., Harding L.K., Jeukendrup A.E. (2005). Oxidation of combined ingestion of glucose and sucrose during exercise. Metabolism.

[44] Jentjens R.L.P.G., Jeukendrup A.E. (2005). High rates of exogenous carbohydrate oxidation from a mixture of glucose and fructose ingested during prolonged cycling exercise. Br. J. Nutr..

[45] Wallis G.A., Yeo S.E., Blannin A.K., Jeukendrup A.E. (2007). Dose-response effects of ingested carbohydrate on exercise metabolism in women. Med. Sci. Sports Exerc..

[46] Rehrer N.J., van Kemenade M., Meester W., Brouns F., Saris W.H. (1992). Gastrointestinal complaints in relation to dietary intake in triathletes. Int. J. Sport Nutr..

[47] Peters H.P.F., Wiersma J.W.C., Koerselman J., Akkermans L.M.A., Bol E., Mosterd W.L., De Vries W.R. (2000). The effect of a sports drink on gastroesophageal reflux during a run-bike-run test. Int. J. Sports Med..

[48] Howlett K.F., Watt M.J., Hargreaves M., Febbraio M.A. (2003). Regulation of glucose kinetics during intense exercise in humans: Effects of α- and β-adrenergic blockade. Metabolism.

[49] Davinelli S., Intrieri M., Ali S., Righetti S., Mondazzi L., Scapagnini G., Corbi G. (2023). Omega-3 index and AA/EPA ratio as biomarkers of running-related injuries: An observational study in recreational runners. Eur. J. Sport Sci..

[50] Davinelli S., Medoro A., Intrieri M., Saso L., Scapagnini G., Kang J.X. (2022). Targeting NRF2–KEAP1 axis by Omega-3 fatty acids and their derivatives: Emerging opportunities against aging and diseases. Free Radic. Biol. Med..

[51] Janse De Jonge X.A.K. (2003). Effects of the menstrual cycle on exercise performance. Sports Med..

