# Supplementary material for: Effects of Maltodextrin–Fructose Supplementation on Inflammatory Biomarkers and Lipidomic Profile Following Endurance Running: A Randomized Placebo-Controlled Cross-Over Trial
Source: Nutrients. 2024 Sep 12;16(18):3078. doi: 10.3390/nu16183078 (PMC11434986; doi:10.3390/nu16183078)
Supplement: Supplementary file 1 [file nutrients-16-03078-s001.zip › nutrients-3172868-supplementary.pdf]

Supplementary material

Table S1. Baseline characteristics of the enrolled athletes.

n	**Overall** 29	**Excluded** 3	**Included** 26	***p*-value***
Age (y)	33 (25, 44)	49 (43, 53)	32 (24.25, 40)	0.053
Sex, n (%)				1.000
Male	25 (86.2)	3 (100.0)	22 (84.6)	
Female	4 (13.8)	0 (0.0)	4 (15.4)	
Weight (Kg)	65.30±8.57	68.40±10.28	64.94±8.52	0.518
Likert scale	3.03 ± 1.64	1.67± 1.53	3.19±1.60	0.128
VO_{2bas} (ml/Kg*min)	6.34±0.81	6.67±0.45	6.30±0.84	0.465
VO_{2max} (ml/Kg*min)	61.33±5.38	56.93±4.07	61.84±5.33	0.137
vVO_{2max} (km/h)	17.64±1.62	16.83±1.20	17.73±1.66	0.374
VT1 (km/h)	15.06±1.50	14.73±1.05	15.10±1.55	0.699
VT2 (km/h)	16.55±1.44	15.97±1.46	16.62±1.46	0.469

VO_{2bas}=oxygen consumption at rest;VO_{2max}=maximal oxygen consumption; vVO_{2max}=velocity at maximal oxygen consumption; VT1=first ventilatory threshold; VT2=second ventilatory threshold. Results were shown as mean (SD) or median (I-III quartile); **p*-value refers to the comparison between excluded and included patients.

Table S2. Descriptive statistics of interaction effects for all lipidomic parameters.

**Parameter**	**Interaction (time x arm)**
AA	p=0.4790
EPA	p=0.6504
DHA	p=0.8990
ω-3 index	p=0.8310
AA/EPA ratio	p=0.5860

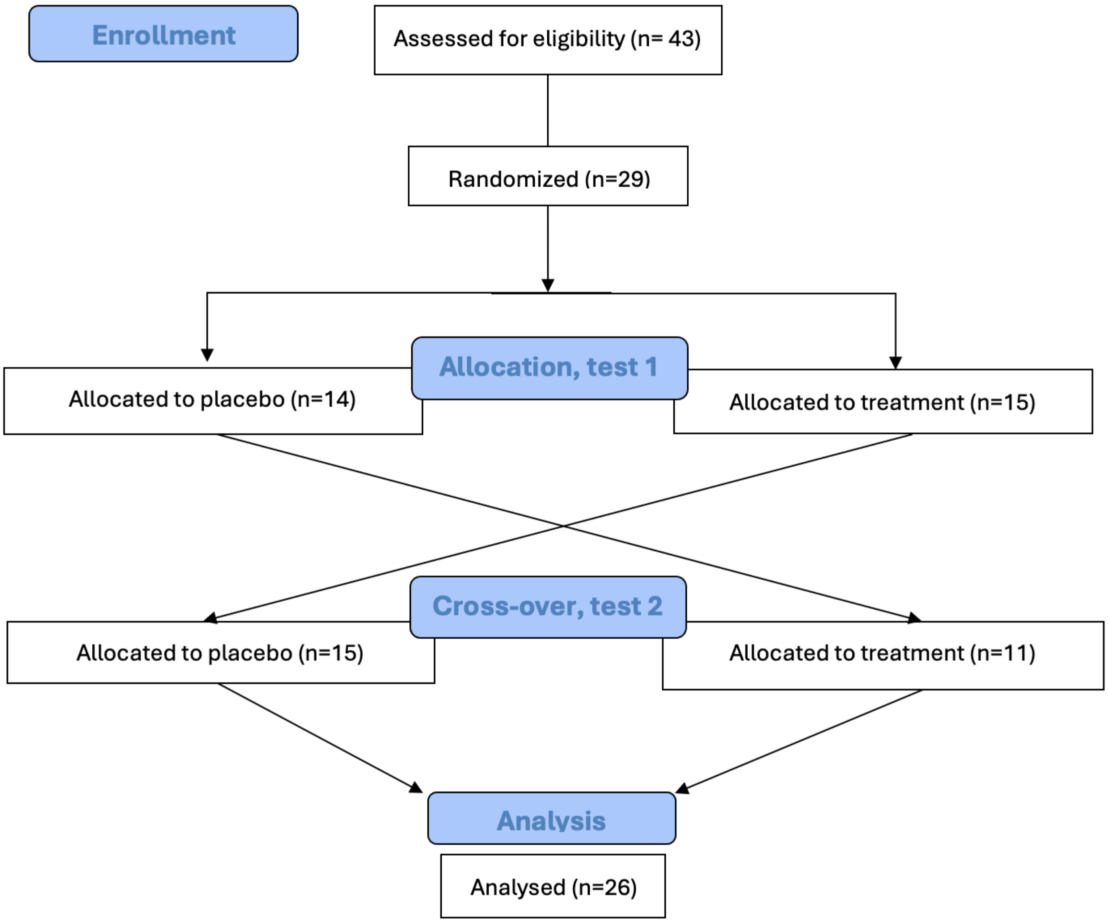

Figure S1. Consort flow diagram depicting the flow of participants through the crossover trial.

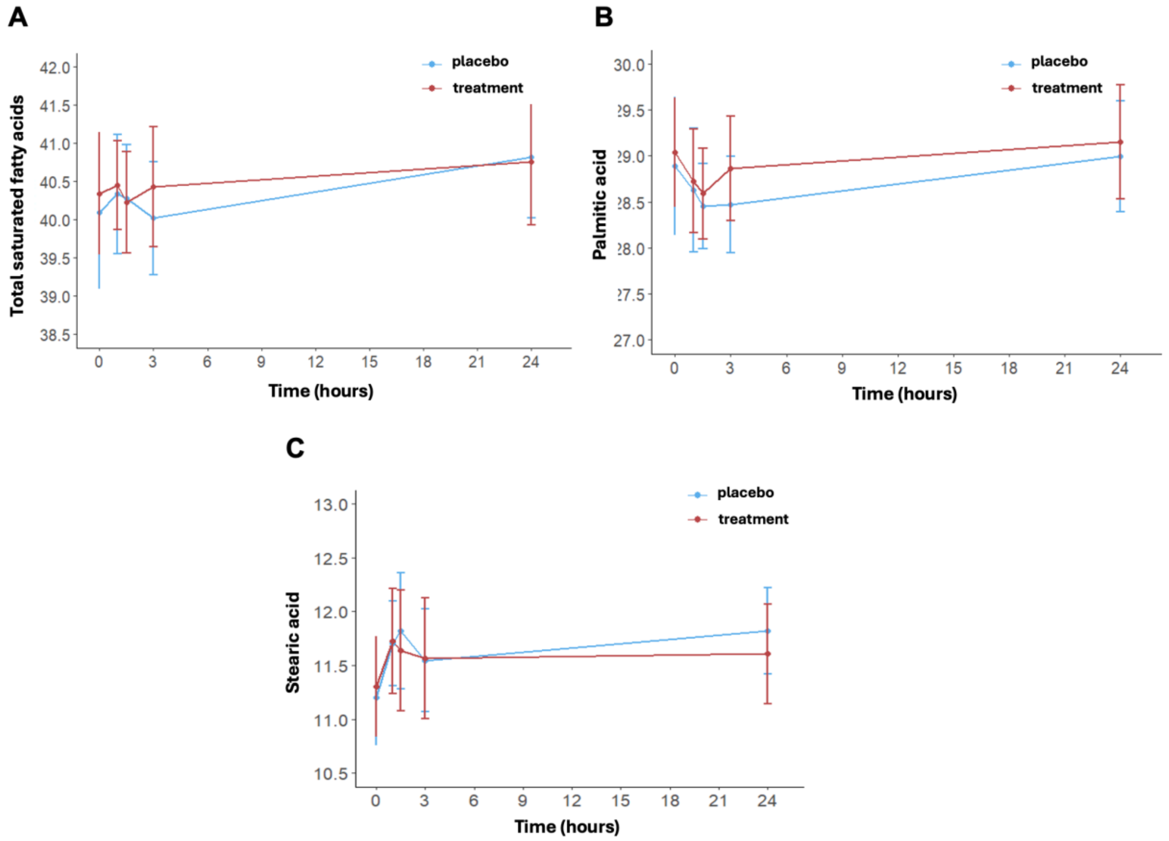

Figure S2. total saturated fatty acids (A) palmitic acid (B) and stearic acid (C) at baseline, post-activity, after 1.5-, 3-, and 24-hours post-running.
